# Mental toughness as a mediator between sports psychological skills and athlete burnout: a buffering model

**DOI:** 10.3389/fpsyg.2025.1677985

**Published:** 2025-10-24

**Authors:** Zhao Zhang, Shaoran Yu

**Affiliations:** ^1^School of Sports Training, Chengdu Sport University, Chengdu, China; ^2^Deyang Vocational College of Technology and Trade, Deyang, China

**Keywords:** mental toughness, athlete burnout, sports psychological skills, mediation analysis, collegiate athletes

## Abstract

**Introduction:**

Athlete burnout is a critical issue in competitive sports, yet the mechanisms linking sports psychological skills to burnout remain under-explored. This study has proposed a mental toughness-mediated buffering model to address this gap.

**Methods:**

Data from 341 collegiate athletes were analyzed using hierarchical regression and Hayes’ PROCESS marco 3.5 (Model 4) with 5000 bootstrap samples for mediation effects.

**Results:**

Key findings have shown that: (1)Sports psychological skills negatively predicted all three burnout dimensions (reduced sense of accomplishment, devaluation, and emotional/physical exhaustion); (2)Mental toughness partially mediated the effects of sports psychological skills on devaluation (*β* = −0.135, 95%CI[−0.249, −0.026]) and reduced sense of accomplishment (*β* = −0.159, 95%CI[−0.261, −0.062]). (3) However, no significant mediation effect was observed for emotional/physical exhaustion, suggesting distinct pathological mechanisms for this dimension.

**Discussion:**

These results have advanced existing burnout theories by demonstrating dimension-specific mediation pathways, highlighting the need for targeted mental toughness interventions to mitigate devaluation and reduced sense of accomplishment in collegiate athletes.

## Highlights

- All three dimensions of sports psychological skills negatively predict burnout, and athletes’ mastery of sport psychology skills could reduce the incidence of burnout.
- Mental toughness mediates the relationship between sports psychological skills and reduced sense of accomplishment, and the buffering model is supported.
- Mental toughness mediates the relationship between sport psychological skills and devaluation, and the buffering model is supported.
- Mental toughness did not play a mediating role between sports psychological skills and emotional/physical exhaustion, and the buffering model did not hold due to athletes’ lack of autonomy and unmet psychological needs.
- Mental toughness provides fine mental cushion and protection for athletes.

## 1 Introduction

While elite athletes like Carmelo Anthony exemplifies resilience in overcoming career-threatening burnout through psychological interventions, the precise mechanism linking sports psychological skills to burnout mitigation remains poorly understood. This study addresses a critical gap by proposing mental toughness as a unifying mediator to investigates whether it mediates the effect of sports psychological skills on each dimension of burnout, and whether these mediation effects are dimension-specific.

Sports psychological skills are a set of trainable mental abilities that athletes must possess in competitive situations and enhance their performance, increase consistency, and achieve greater satisfaction in sports and physical activities (Filion et al., 2021; Mahoney et al., 1987). Mahoney has conceptualized these skills through 6 broad traits: anxiety measurement, concentration, self-confidence, mental preparation, team emphasis, and motivation.

Studies have found that athletes’ sports psychological skills were closely related to their sports performance. Recent research has deepened our understanding of this relationship in several important ways. Meta-analytic evidence of training effects: A systematic review and meta-analysis of sports psychological skills training programs across a wide range of sports reported moderate to large improvements in performance outcomes (Reinebo et al., 2024). Multiple studies across different sports and competition levels report moderate to large correlations between self reported psychological skill scores and objective performance metrics such as speed, accuracy, or competition ranking (Lochbaum et al., 2022). Researchers (Zhang et al., 2024) also reported that combining Acceptance and Commitment Therapy (ACT) with traditional sports psychological skills training enhances athletes’ ability to stay focused under pressure and reduces maladaptive rumination.

As competitive sports tactics and techniques reach peak levels, sports psychological skills have crucial weapons for athletes seeking self-breakthrough and excellence (Qiu and Yao, 2007). Psychological training now forms an integral part of the modern scientific sports training system, alongside physical, technical, and tactical training. In China, psychological training has gained widespread recognition. Increasingly, sports psychologists participate in the training and competition programs of national-level and provincial-level teams, providing systematic psychological services. However, coaches’ and athletes’ primary expectations for this training often center on overcoming immediate mental issues, improving mental states, and facilitating a return to normal training and competition. In contrast, the main purpose of psychological training should be to help athletes proactively master psychological adjustment techniques, enhance their sports psychological skills, and foster psychological development, thereby enabling them to flexibly cope with competitive pressure and unexpected events. Existing researches predominantly focus on how psychological skills improve sports performance, conclusions largely consistent with sports practice common sense, limiting innovation in this area. Chinese sports psychologists have gradually recognized that the focus of sports psychological skills training should prioritize athletes’ long-term well-being and self-development, rather than merely maintaining good training states or achieving peak performance (Zhang and Zhang, 2011). Therefore, exploring the role of sports psychological skills in enhancing athletes’ comprehensive psychological abilities—particularly their capacity to manage long-term career challenges such as burnout—represents an emerging and worthwhile research direction.

Burnout refers to the phenomenon where mental function declines below its original level due to the continuous depletion of psychological resources under sustained pressure, without timely replenishment. It manifests in emotional, cognitive, motivational, behavioral, and physiological dimensions (Zhang and Mao, 2007). Athlete burnout is a psychophysiological syndrome that emerges when the continuous, high intensity demands of training, competition, and related life domains exhaust an athlete’s psychological and physiological resources faster than they can be replenished (Cai et al., 2025; Dišlere et al., 2025). Researcher (Maslach, 2003) typically conceptualize burnout using a three-factor model: reduced sense of accomplishment (core features: reduced intrinsic drive, loss of enjoyment, questioning “why continue”), emotional/physical exhaustion (core features: persistent fatigue, loss of energy, feeling “drained” even after rest), and devaluation (core features: the diminishment or denial of the value of sports itself).

Studies indicate that training/competition satisfaction and sports motivation are significant predictors of burnout (Zhang, 2008). Prolonged burnout adversely affect training, competition, and long-term career development (Glandorf et al., 2022; Woods et al., 2020). Consequently, Zhang (2010) has argued that the triggering factors and management of burnout warrant greater attention. Researchers (Woods et al., 2025) suggests that a lack of autonomy may be a key antecedent. Enhancing athletes’ autonomy during training, such as granting them decision-making power and fostering responsibility, can help maintain interest and motivation, thereby reducing burnout (Cipriano, 2024; Jin et al., 2022). Although a lack of autonomy is an established antecedent of burnout, the protective mechanism of sports psychological skills against burnout lacks a clear explanation.

Crucially, mental toughness is a psychological construct that enables athletes to stay determined, focused, confident and calm when faced with high-pressure or adverse situations. It reflects the ability to maintain or even improve performance despite stress, setbacks, or fatigue (Hsieh et al., 2024). It enables athletes to maintain focus, execute skills, and make optimal decisions when stakes are high; Facilitates rapid recovery from injury, failure, or poor performance, turning adversity into learning; and acts as a protective factor: athletes high in mental toughness report lower emotional exhaustion and sport devaluation, thereby reducing burnout risk (Poulus et al., 2024). It also develops through sports psychological skills training (Pocius and Malinauskas, 2024).

Mental toughness may mediates between sports psychological skills and burnout. This mediation functions through its core stress-regulating mechanism, operating via two pathways: (1) resource conservation, and (2) cognitive restructuring.

Through its dual pathways of conserving cognitive resources and restructuring stress appraisals, mental toughness transforms sports psychological skills into a robust buffer against athletes’ burnout.

While prior studies have linked sports psychological skills to performance outcomes (Kalén et al., 2021), their role in mitigating burnout—particularly through mental tough mechanisms—remains unclear. Existing literatures predominantly focus on burnout antecedents (e.g., training load, motivation), with limited attention to how sports psychological skills interact with mental toughness to buffer burnout.

Current literature bifurcates into: (1) Studies on sports psychological skills and performance (e.g., Latinjak et al., 2023), and (2) Researches on autonomy and burnout (e.g., Woods et al., 2025), leaving the buffering pathway unexplored. No integrated model examines whether mental toughness mediates the relationship between sports psychological skills and burnout.

This study pioneers in verifying mental toughness’s mediation between sports psychological skills and burnout—a mechanistic insight absent in prior literature that predominantly treat burnout as a unitary construct.

### 1.1 Research hypothesis

*Hypothesis H1*: Sports psychological skills negatively predict burnout in athletes.

**Figure 1:**
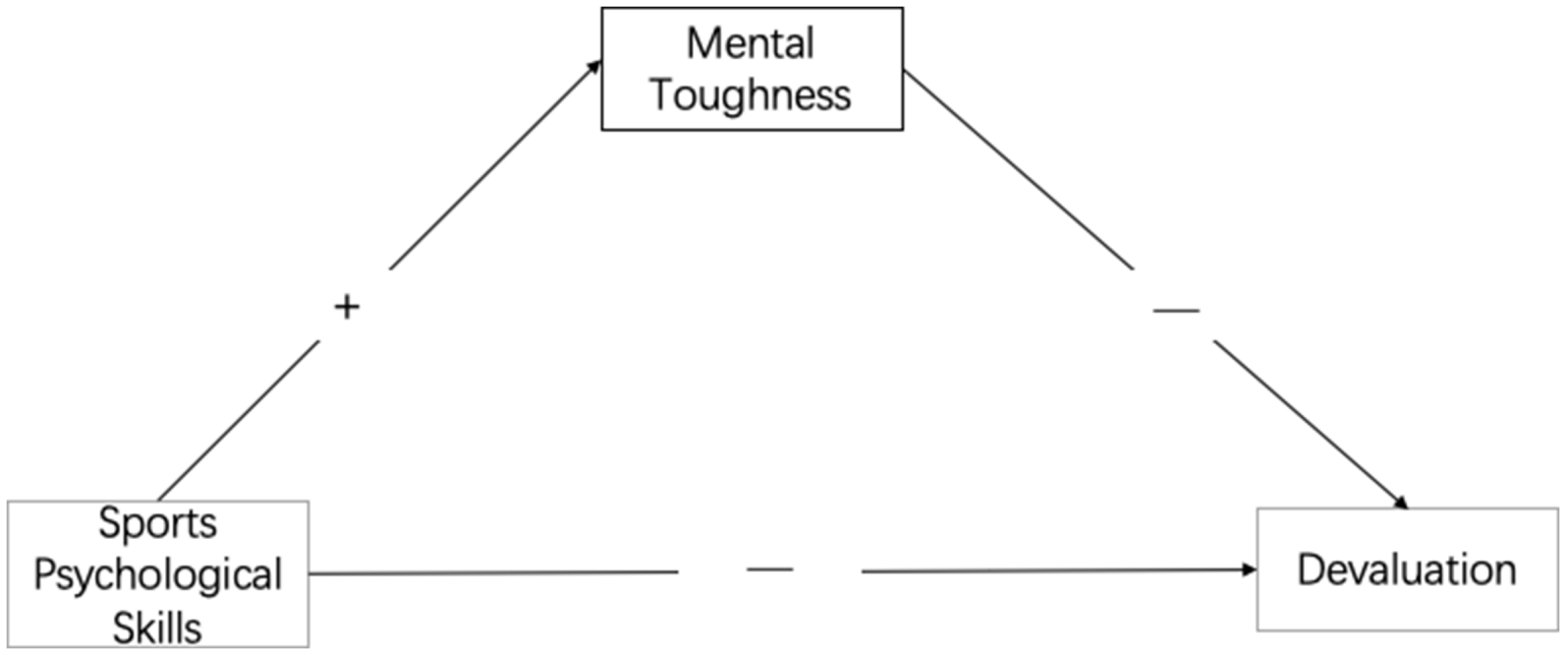
The mental toughness buffering model to control devaluation.

**Figure 2:**
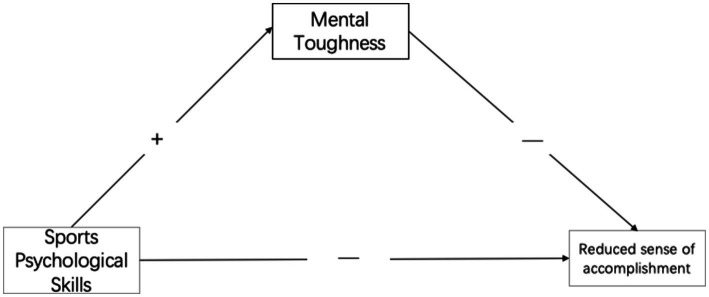
The mental toughness buffering model to control reduced sense of accomplishment.

**Figure 3:**
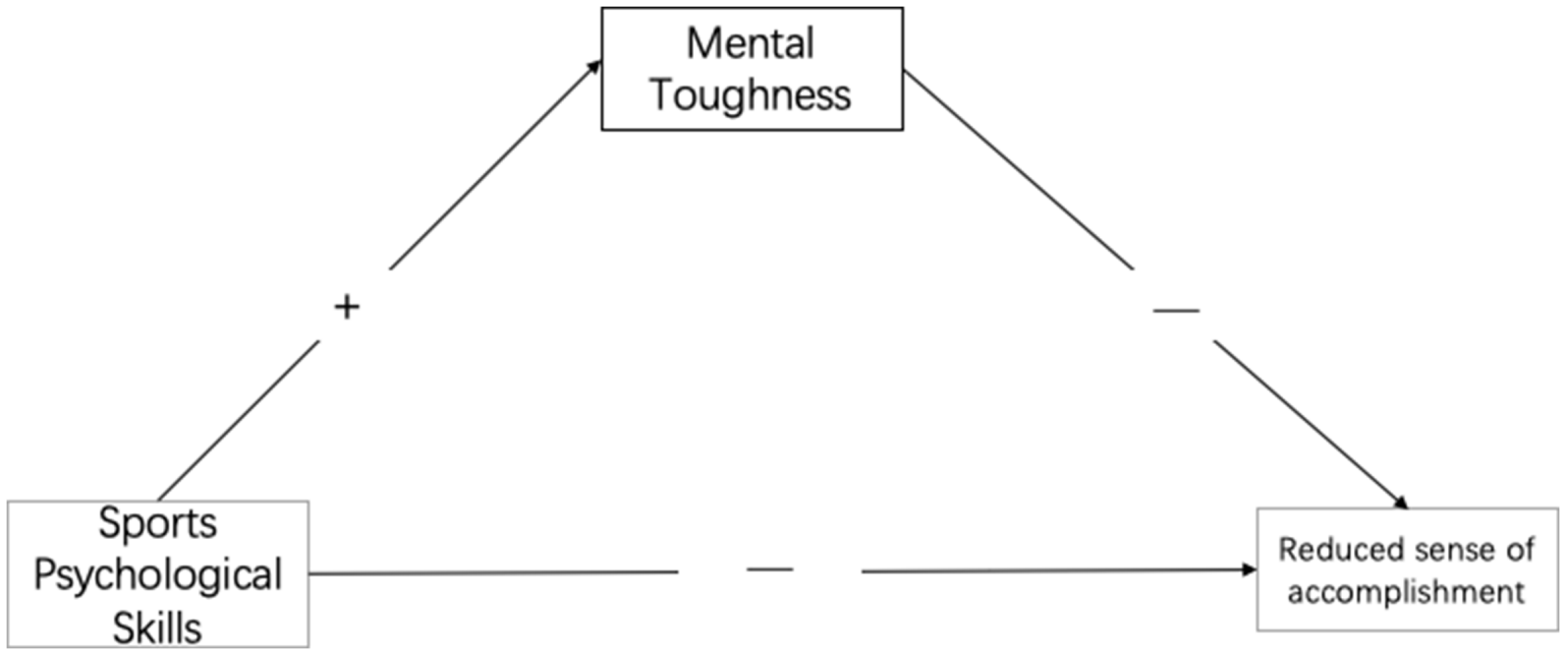
The mental toughness buffering model to control emotional/physical exhaustion.

*Hypothesis H2-1*: Mental toughness mediates between sports psychological skills and devaluation;

*Hypothesis H2-2*: Mental toughness mediates between sports psychological skills and reduced sense of accomplishment;

*Hypothesis H2-3*: Mental toughness mediates between sports psychological skills and emotional/physical exhaustion (see Figures 1–3).

This model advances burnout theory by testing a cognitive-motivational pathway distinct from physiological exhaustion drivers, informing targeted interventions for sustainable athletic development.

### 1.2 Research objectives

#### 1.2.1 Primary objective

To examine whether mental toughness mediates the relationship between sports psychological skills and athlete burnout, and to test a buffering model in which mental toughness serves as a protective mechanism against burnout among collegiate athletes.

#### 1.2.2 Secondary objectives

To investigate the direct predictive effects of sports psychological skills on each of the three burnout dimensions: emotional/physical exhaustion, reduced sense of accomplishment, and devaluation (H1).

To assess whether mental toughness mediates the relationship between sports psychological skills and: devaluation (H2-1); reduced sense of accomplishment (H2-2); emotional/physical exhaustion (H2-3).

To provide empirical evidence for designing targeted psychological interventions aimed at reducing burnout through enhanced mental toughness and sports psychological skills.

## 2 Materials and methods

### 2.1 Participants

Employing a cross-sectional design, we recruited a sample of collegiate athletes. Participants were selected via snowball sampling across multiple universities in China, targeting individuals aged 17–22 years who were actively competing at the provincial level or above. Data collection occurred at a single time point using an online survey during January 14, 2025, to February 7, 2025. Each participant filled out the survey questionnaires online according to the instruction of the scale. The survey utilized validated Chinese versions of the relevant scales, and informed consent was obtained prior to participation, for minor participants (*n* = 8, aged 17), written informed consent was secured from their parents or legal guardians. To mitigate common method bias, we employed procedural remedies (e.g., anonymity, reverse-scored items) alongside Harman’s single-factor test.

A total of 362 questionnaires were distributed, 21 copies were found to have incomplete or missing data, and 341 valid questionnaires were recovered, with an effective recovery rate of 94.2%. Among them, 7 (2.1%) athletes at the national master level, 93 (27.3%) were first-level, 240 (70.4%) were second-level and 1 (3%) was third-level; 222 (65.1%) athletes were male and 119 (34.9%) were female; the average age of the subjects was 20.76 years old (SD = 1.06 year) and the average training period was 6.6 years (SD = 2.45 years). The sports covered by the survey respondents included track and field, swimming, basketball, volleyball, badminton, tennis, taekwondo, weightlifting, karate, water polo, rhythmic gymnastics, kayaking and other individual/group sports.

The sample covered 12 + sports reflecting the multi-sport landscape of Chinese collegiate athletes. This diversity mitigated sport-specific bias. Participants were recruited from multiple universities across China (via snowball sampling), reducing regional concentration risks. Athletes spanned provincial to national levels (e.g., 27.3% first-level, 70.4% second-level), aligning with the tiered competitive structure common in Chinese collegiate sports systems. Age range and training experience reflected typical collegiate athlete profiles in China, where early specialization was prevalent.

### 2.2 Instruments

The survey included three validated instruments, selected based on conceptual alignment with core constructs and psychometric robustness in athletic populations:

#### 2.2.1 Psychological skill inventory for sports (PSIS)

The scale was developed by American psychologists (Mahoney et al., 1987) and revised to a Chinese edition (Zhang and Mao, 2004). The PSIS was selected for three reasons: (1) Its multidimensional structure aligned with our theoretical model; (2) The validated Chinese version demonstrated adequate reliability and cross-cultural applicability; (3) It efficiently captured trainable skills relevant to intervention design. There are 45 items in this scale which are decomposed into 6 subscales and definitions: Anxiety control (AX):ability to manage worry and physiological arousal; Concentration (CC): capacity to maintain focus on task-relevant cues; Confidence (CF):belief in one’s ability to perform under pressure; Mental preparation (MP): use of imagery, self-talk, and routine planning; Motivation (MV)drive to train and compete, goal orientation; and Team emphasis (TM): importance placed on team cohesion and collective goals. Specific items such as “I try not to think about my movements 24 h before the games,” “In most competitions, I believe I will perform better.” A 5-point Likert is used, with a scale of 1 to 5 ranging from “completely disagree” to “completely agree.” The higher the total score and the score of each dimension of the scale, the better the sports psychological skills. In this study, the PSIS’s Cronbach’s *α* coefficient was 0.82, and Cronbach’s α coefficient of the six dimensions were 0.72(AX), 0.71(CC), 0.81(CF), 0.66(MV),. 74(TM), and 0.61(MP).

#### 2.2.2 Sports mental toughness questionnaire (SMTQ)

The questionnaire was developed by Sheard et al. (2009), and revised to a Chinese edition (Wang et al., 2013). The questionnaire directly captured the resilience dimensions theorized to buffer burnout (Gustafsson et al., 2017). It aligned with our aim to examine how mental toughness protect against burnout. There are 12 items in total, such as “I can perform well under pressure,” “I am committed to completing my personal tasks.” A 5-point Likert is used, with a scale of 1 to 5 ranging from “completely disagree” to “completely agree.” The SMTQ’s Cronbach’s *α* coefficient was 0.75.

#### 2.2.3 Athlete burnout questionnaire (ABQ)

The questionnaire was developed by Raedeke and Smith (2001), revised to a Chinese edition (Zhang and Mao, 2004). The questionnaire is the gold standard for operationalizing athlete-specific burnout, distinct from occupational measures (Eklund and DeFreese, 2015; Grugan et al., 2024). It contains 15 items and is divided into 3 dimensions: emotional/physical exhaustion (such as “I was extremely tired during training”), reduced sense of accomplishment (such as “I have the joy of being successful in sports”), devaluation (such as “I cannot play as intently as I used to”). A 5-point Likert is used, with a scale of 1 to 5 ranging from “none” to “always.” In this study, the ABQ’s Cronbach’s *α* coefficient was 0.86, and Cronbach’s α coefficient of the three dimensions were 0.78 (emotional/physical exhaustion), 0.77 (devaluation), and 0.58 (reduced sense of accomplishment).

### 2.3 Data process

We used SPSS 19.0 and Model 4 of the PROCESS marco (Model 4) (Hayes, 2017) to organize and analyze the data. Statistical methods including reliability tests, descriptive statistics, correlation analysis, analysis of variance, and Bootstrap analysis of mediating effects were employed. Hierarchical regression controlled for gender, age, and training years. Mediation effects were tested using Hayes’ PROCESS marco (Model 4) with 5,000 bootstrap samples. The significance level was set at α = 0.05.

## 3 Results

### 3.1 Common method bias

We used Harman’s one-factor test to diagnose the data: exploratory factor analysis was performed on all observed variables in this study, forcing only one common factor to be drawn and no rotation. It was found that the variance explained ratio of the extracted first common factor was 14.758%, which was lower than the 40% standard proposed by Podsakoff et al. (2003).

### 3.2 Sports psychological skills level of collegiate athletes

The average scores of each dimension of sports psychological skills from high to low were 3.532 (confidence), 3.504 (motivation), 3.326 (psychological preparation), 3.266 (team emphasis), 3.232 (anxiety measurement), and 3.201 (concentration). The average score was 3.348. Only the anxiety measurement exceeded the norm group, and the sports psychological skills’ level of collegiate athletes was at a moderately low level. This study also examined differences in sports psychological skills by gender, sport level, event type, and years of training. The results have shown that the main effect of gender was significant (*F* = 3.798, *p* = 0.053, partial *η*_p_^2^ = 0.020, small effect size), and the total score of sports psychological skills in males (*M* = 3.410) was significantly higher than that in females (*M* = 3.233). The main effect of sport level (*F* = 0.551, *p* = 0.648, partial *η*_p_^2^ = 0.009), sports type (*F* = 0.056, *p* = 0.813, partial *η*_p_^2^ = 0.000) and training period (*F* = 0.498, *p* = 0.923, partial *η*_p_^2^ = 0.034) were not statistically significant.

### 3.3 Correlation analysis of sports psychological skills, mental toughness and burnout

Correlation analysis showed that the emotional/physical exhaustion dimension of sports-induced burnout was negatively correlated with sports psychological skills and their dimensions, anxiety measurement, concentration, self-confidence, and team emphasis; reduced sense of accomplishment was negatively correlated with sports psychological skills and its dimensions; devaluation was negatively correlated with all dimensions of sports psychological skills except psychological preparation. Mental toughness was negatively correlated with all dimensions of burnout. In addition to team emphasis, all dimensions of sports psychological skills were positively correlated with mental toughness (see Table 1).

**Table 1:** Means, standard deviations, and correlation coefficients among sports psychological skills, mental toughness, and burnout (*n* = 341).

Variables	1	2	3	4	5	6	7	8	9	10	11
1. Anxiety measurement	—										
2. Concentration	0.480^**^	—									
3. Confidence	0.690^**^	0.434^**^	—								
4. Motivation	0.202^**^	0.050	0.349^**^	—							
5. Team emphasis	0.182^**^	0.072	0.157^**^	0.182^**^	—						
6. Psychological preparation	0.065	−0.051	0.193^**^	0.424^**^	0.067	—					
7. Mental toughness	0.661^**^	0.402^**^	0.713^**^	0.368^**^	0.091	0.192^**^	—				
8. Emotional/physical exhaustion	−0.283^**^	−0.260^**^	−0.226^**^	−0.006	−0.202^**^	0.104	−0.246^**^	—			
9. Reduced sense of accomplishment	−0.398^**^	−0.225^**^	−0.442^**^	−0.285^**^	−0.214^**^	−0.119^*^	−0.452^**^	0.519^**^	—		
10. Devaluation	−0.304^**^	−0.192^**^	−0.290^**^	−0.154^**^	−0.216^**^	0.086	−0.309^**^	0.736^**^	0.573^**^	—	
11. Sports psychological skills	0.778^**^	0.518^**^	0.854^**^	0.594^**^	0.379^**^	0.440^**^	0.718^**^	−0.241^**^	−0.480^**^	−0.304^**^	—
MEAN	3.232	3.201	3.532	3.504	3.266	3.326	3.233	2.597	2.503	2.832	3.348
Standard deviation	0.511	0.476	0.692	0.567	0.419	0.622	0.529	0.889	0.714	0.874	0.353

^*^p < 0.05, ^**^p < 0.01, ^***^p < 0.001.

### 3.4 Regression of sports psychological skills on burnout

Hierarchical regression was used to examine whether sport psychological skills had an incremental contribution to burnout controlled for demographic variables (gender, age, sports events, training years, and skill level). The results showed that sports psychological skills negatively predict emotional/physical exhaustion (*β* = −0.293, *p* = 0.000), devaluation (*β* = −0.339, *p* = 0.000), and reduced sense of accomplishment (*β* = −0.517, *p* = 0.000), explaining 7.8, 10.6, and 24.5% of the variance, respectively, (see Table 2). In summary, H1 is supported.

**Table 2:** The hierarchical regression results of burnout’s predictors.

Variables	Emotional/physical exhaustion		Devaluation		Reduced sense of accomplishment	
	*β*	*t*	*β*	*t*	*β*	*t*
1. Gender	−0.095	−1.686	−0.019	−0.335	−0.004	−0.069
Age	−0.014	−0.250	0.067	1.220	0.042	0.760
Sport events	0.087	1.606	0.142^*^	2.628	0.138^*^	2.545
Training years	0.023	0.416	−0.029	−0.523	−0.051	−0.939
Skill level	−0.049	−0.871	−0.009	−0.157	0.056	1.001
2. Sports psychological skills	−0.293^***^	−5.372	−0.339^***^	−6.347	−0.517^***^	−10.582
*R* ^2^	0.096		0.132		0.274	
ΔR^2^	0.078		0.106		0.245	
*F*	5.890^***^		8.402^***^		20.850^***^	

^*^p < 0.05, ^**^p < 0.01, ^***^p < 0.001.

### 3.5 The mediating effect of mental toughness on the relationship between sports psychological skills and burnout

The interval estimation was used to verify the mediating effect. According to researchers (Hayes and Scharkow, 2013; Wen and Ye, 2014), the basic idea of this method was to perform re-sampling with put-back within the original data, and to draw equal sample data to test the mediating effect. We took sports psychological skills as the independent variable (X), mental toughness as the mediating variable (M), and devaluation, reduced sense of accomplishment, emotional/physical exhaustion the dependent variable respectively (Y). Hierarchical regression and bootstrap analyses (5,000 samples) revealed a divergent mediation pattern across burnout dimensions, directly testing H2-1, H2-2, H2-3.

For devaluation and reduced sense of accomplishment, mental toughness showed significant partial mediation:(1) Devaluation: *ab* = −0.135, 95%CI [−0.249, −0.026], 44.4% of the total effect, H2-1 was supported. (2) Reduced sense of accomplishment: *ab* = −0.159, 95%CI [−0.261, −0.062], 33.1% of total effect, H2-2 was supported.

**Figure 4:**
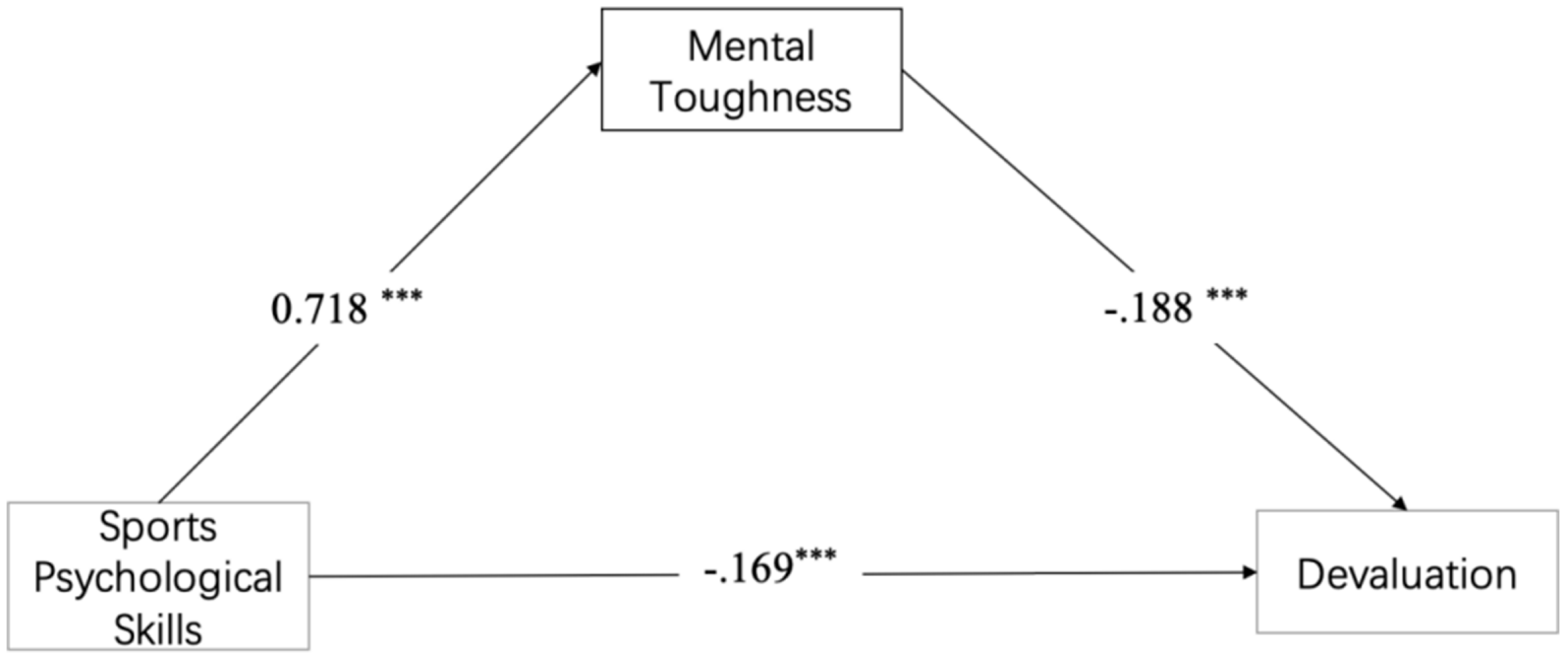
Mediation model showed the indirect effect of sports psychological skills on devaluation via mental toughness.

**Figure 5:**
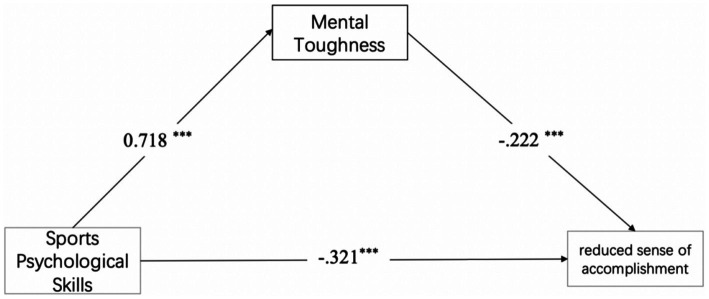
Mediation model showed the indirect effect of sports psychological skills on reduced sense of accomplishment via mental toughness.

**Figure 6:**
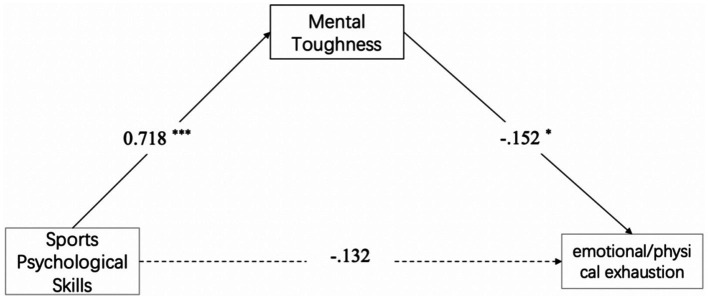
The mediating effect model of mental toughness between sports psychological skills and emotional/physical exhaustion. ^*^p < 0.05, ^**^p < 0.01, ^***^p < 0.001.

For emotional/physical exhaustion, no mediation emerged: *ab* = −0.109, 95%CI [−0.232, 0.011], H2-3 was not supported consequently (see Table 3 and Figures 4–6).

**Table 3:** Bootstrap analysis of mental toughness mediation across burnout dimensions.

Paths	Effects	Boot SE	95% CI		Relative mediation effect
			Lower	Upper	
Devaluation					
Total effect (*c*)	−0.304	0.147	−1.149	−0.573	
Direct effect (*c’*)	−0.169	0.209	−0.889	−0.067	55.59%
Indirect effect (*ab*)	−0.135	0.160	−0.249	−0.026	44.4%
Reduced sense of accomplishment					
Total effect (*c*)	−0.480	0.135	−1.625	−1.094	
Direct effect (*c’*)	−0.321	0.191	−1.285	−0.533	66.9%
Indirect effect (*ab*)	−0.159	0.050	−0.261	−0.062	33.1%
Emotional/physical exhaustion					
Total effect (*c*)	−0.241	0.149	−0.976	−0.388	
Direct effect (*c’*)	−0.132	0.214	−0.793	0.047	54.8%
Indirect effect (*ab*)	−0.109	0.062	−0.232	0.011	45.2%

## 4 Discussion

This study successfully achieved its primary objective by constructing and validating a mental toughness-mediated buffering model, which elucidates a key psychological mechanism through which sports psychological skills mitigate athlete burnout. The mediation effect analysis revealed that mental toughness partially mediates the effect between sports psychological skills and burnout, suggesting that these skills help reduce burnout partly through the enhancement of mental toughness. However, comparative analysis revealed inconsistencies in mental toughness’s role across the three burnout dimensions, pointing to a dimension-specific buffering mechanism.

First, confirming our first hypothesis (H1), sports psychological skills negatively predicted all three dimensions of burnout. That is, a higher level of sports psychological skills is associated with a lower degree of burnout, indicating that these skills are conducive to coping with and improving this condition. This finding aligns with Smith’s (1986) cognitive-affective model of athletic burnout, which posits that burnout is more likely to occur when athletes cannot cope with situational demands and perceive stress as a threat. Therefore, sufficient coping resources make burnout less likely. Corroborating this, researchers (He et al., 2014) have found a positive correlation between sports psychological skills and coping styles. This indicates that athletes with superior sports psychological skills are more likely to adopt positive coping strategies to manage and adapt to stressful stimuli in training and competition, thereby maintaining a better mental state and preventing burnout.

Further analysis reveal that sports psychological skills had a greater impact on reduced sense of accomplishment than the other two dimensions. A reduced sense of accomplishment, the efficacy component of burnout, relates to an athlete’s perceived skills and abilities, often stemming from an inability to achieve personal goals. According to Psychological Capital Theory (Luthans et al., 2006), positive psychological capital—comprising self-efficacy, optimism, hope, and resilience—is a critical res + ource for individuals to cope with burnout. Numerous studies have found that athletes’ positive psychological capital is beneficial against burnout (Lin et al., 2022; Santana et al., 2023; Strümpfer, 2003). In our study, self-confidence—a core dimension of sports psychological skills—demonstrated a higher correlation with a reduced sense of accomplishment than other components, which strongly corroborates the psychological capital theory. This insight suggests that by continuously developing athletes’ positive psychological capital through psychological skills training, sports psychologists can effectively reduce the occurrence of a reduced sense of accomplishment.

The central finding of this research, addressing our primary objective, is the dimension-specific mediating role of mental toughness. It partially mediated the effects of sports psychological skills on devaluation (H2-1) and reduced sense of accomplishment (H2-2), thereby supporting the buffering model for these two motivational-cognitive dimensions. However, no significant mediation was found for emotional/physical exhaustion (H2-3). This divergent result provides strong empirical support for the proposed buffering model for devaluation and reduced sense of accomplishment while simultaneously revealing its limits regarding the physiological exhaustion dimension.

The successful mediation for devaluation and reduced sense of accomplishment demonstrates that the protective effect of sports psychological skills operates substantially through enhanced mental toughness. This supports the core theoretical premise that mental toughness, cultivated through psychological skills training, provides athletes with the cognitive-motivational resources to persevere. When confronting failure or stagnation, athletes with higher mental toughness are better equipped to maintain their commitment (thus countering devaluation) and to reframe setbacks as growth opportunities (thus preserving their sense of accomplishment). This cognitive-affective pathway effectively buffers the motivational erosion defining these two burnout dimensions, corroborating previous claims that mental toughness is pivotal for sustaining long-term engagement in sport.

Conversely, the absence of mediation for emotional/physical exhaustion unveils a critical physiological-psychological dissociation in burnout pathways. As the depletion component, emotional/physical exhaustion is closely tied to the intense demands of training and competition. Its emergence is less controllable through cognitive means and is significantly influenced by objective stressors (Ryan and Deci, 2000). This lack of mediation aligns with Self-Determination Theory (Ryan and Deci, 2000). We posit that emotional/physical exhaustion predominantly stems from thwarted basic psychological needs—particularly autonomy—a deficit less amenable to remediation through psychological skills interventions alone. Evidence converges from several angles: Studies show that autonomy and competence needs negatively predict emotional/physical exhaustion (Lonsdale et al., 2009), yet autonomy-supportive interventions do not necessarily increase mental toughness (Mahoney et al., 2016), indicating distinct mechanisms. Furthermore, physiological studies confirm that emotional/physical exhaustion is linked to hypothalamic–pituitary–adrenal (HPA) axis dysfunction, manifesting as an elevated cortisol awakening response (Kadooka et al., 2024). This physiological dysregulation operates independently of cognitive regulatory mechanisms like mental toughness. While sports psychological skills optimize cognitive resources, they cannot replenish physiological resources drained by overtraining or compensate for structural autonomy deficits. Therefore, when athletes lack control over demanding training schedules, mental toughness may delay but cannot prevent eventual energy bankruptcy, explaining its failure to mediate this specific burnout dimension.

Athletes’ mental burnout directly impacts their sports performance and also determines their mental health levels and career development (Glandorf et al., 2025). These findings have direct practical implications. For the motivational dimensions of reduced sense of accomplishment and devaluation, interventions should leverage the mediating role of mental toughness. This involves integrating sports psychological skills training into regular practice to build psychological capital (e.g., confidence, motivation) (Xiong and Ye, 2014) and conducting regular mental toughness screenings for personalized interventions. Within the context of China’s Sports-Education Integration policy, this study provides an empirical basis for strengthening psychological support for athletes. For example, institutional reforms could allow athletes to exchange competition achievements for general education credits, alleviating the reduced sense of accomplishment caused by academic training conflicts (Popa-Velea et al., 2025). Finally, integrate sports psychological skills training into regular technical-tactical and physical training (Kusuma et al., 2024). Combining this with sport-specific characteristics will enhance athletes’ sports psychological skills levels (Talha, 2023). Thoughtfully explore the role of sports psychological skills in athletes’ overall psychological capabilities, ultimately helping them address long-term challenges throughout their careers. For emotional/physical exhaustion, which operates on a different pathway, interventions must go beyond psychological training. These should include autonomy-supportive strategies like athlete-co-designed training periodization, physiological monitoring (e.g., cortisol, heart rate variability) to objectively manage training loads, and institutional policies that ensure adequate recovery.

## 5 Limitations and future research

### 5.1 Limitations

Despite its contributions, this study has several limitations that should be considered when interpreting the results. First, the cross-sectional design prevents the establishment of causal relationships. While our model proposes that sports psychological skills enhance mental toughness to reduce burnout, it is plausible that athletes experiencing less burnout subsequently report higher mental toughness and better sports psychological skills. Second, the reliance on self-report measures may introduce common method variance, although Harman’s single-factor test indicated that this was not a severe issue in our data. Third, we treated sports psychological skills as a holistic construct; thus, the unique predictive power and potential mediating pathways of its specific sub-dimensions (e.g., confidence vs. concentration) on different burnout facets remain unexplored. Finally, the generalizability of our findings may be constrained by the specific demographic and cultural context of Chinese collegiate athletes. The influence of China’s unique sports-education system and collectivist culture on these psychological constructs warrant caution when applying these results to other populations.

### 5.2 Future research directions

Building on these findings and limitations, future research should pursue several promising avenues. To address causality, longitudinal or experimental designs are essential (Wang et al., 2025). For instance, cross-lagged panel models tracking athletes across a competitive season could test the temporal precedence of sports psychological skills on mental toughness and burnout. Intervention studies that systematically train sports psychological skills could provide direct evidence for their role in fostering mental toughness and reducing burnout.

Furthermore, investigating additional mediating and moderating variables will provide a more nuanced understanding of the burnout process. Potential mediators such as self-efficacy, self-control, or basic psychological needs satisfaction (autonomy, competence, relatedness) could explain the residual direct effects we observed. Moderators such as gender, sport type (individual vs. team), or coaching style should be explicitly tested to identify the boundary conditions of the buffering model. Future studies should intentionally oversample specific subgroups (e.g., female combat-sport athletes) for this purpose.

Specifically regarding the elusive nature of emotional/physical exhaustion, a multi-method approach is critical. Future studies should integrate psychological scales with physiological biomarkers (e.g., cortisol awakening response, heart rate variability) and objective training load data to triangulate the physiological and psychological antecedents of exhaustion. Finally, qualitative research could offer rich insights into the lived experience of athletes for whom mental toughness “fails” to buffer against exhaustion, exploring the interplay between autonomy, coaching demands, and physiological depletion in real-world settings. For example, does parental expectation pressure alter mental toughness mechanisms in China, as suggested by findings that parental affection and success expectations increased student-athletes’ burnout (Saarinen et al., 2025).

## 6 Conclusion

In conclusion, this study successfully achieved its primary objective by constructing and validating a mental toughness-mediated buffering model, which elucidates a key psychological mechanism through which sports psychological skills mitigate athlete burnout. The main conclusions are as follows:

First, and most critically, the core finding of this research is the dimension-specific mediating role of mental toughness. It functions as a partial mediator in the relationships between sports psychological skills and the motivational-cognitive dimensions of burnout—namely, reduced sense of accomplishment and devaluation. This confirms that part of the beneficial effect of sports psychological skills is achieved by building athletes’ mental toughness, which in turn acts as a psychological buffer, helping them maintain commitment and a positive self-evaluation in the face of adversity.

Second, the study confirms that sports psychological skills are a significant protective factor against burnout, thereby expanding their recognized role beyond enhancing sports performance to include safeguarding athletes’ long-term career development and psychological well-being.

Third, the absence of mediation for emotional/physical exhaustion delineates the boundary of this cognitive-motivational buffering model. This underscores that this dimension, rooted in physiological depletion and thwarted autonomy, requires intervention strategies that go beyond mental skills and toughness training.

Theoretically, these findings significantly advance burnout theories by demonstrating distinct pathways for its different dimensions, thereby challenging the treatment of burnout as a unitary construct. Practically, this study provides a clear, evidence-based roadmap for interventions: coaches and sports psychologists should integrate sports psychological skills training with autonomy-supportive strategies to holistically address the multifaceted nature of athlete burnout, fostering both sustainable performance and long-term mental health.

## Data Availability

The raw data supporting the conclusions of this article will be made available by the authors, without undue reservation.
